# Evaluación de la sensibilidad a organofosforados en poblaciones de *Aedes aegypti* (L.) (Diptera: Culicidae) del departamento de La Guajira, Colombia

**DOI:** 10.7705/biomedica.6677

**Published:** 2023-06-30

**Authors:** Ronald Maestre-Serrano, Zulibeth Flórez-Rivadeneira, Juan Miguel Castro- Camacho, Linda Ochoa-Bohórquez, Doris Gómez-Camargo, Paula Pareja-Loaiza, Gustavo Ponce-García, Adriana E. Flores

**Affiliations:** 1 Facultad de Ciencias de la Salud, Universidad Libre, seccional Barranquilla, Barranquilla, Colombia Universidad Libre Facultad de Ciencias de la Salud Universidad Libre seccional Barranquilla Barranquilla Colombia; 2 Secretaría de Salud Departamental, Gobernación de La Guajira, Riohacha, Colombia Secretaría de Salud Departamental Gobernación de La Guajira Riohacha Colombia; 3 Facultad de Medicina, Universidad de Cartagena, Cartagena de Indias, Colombia Universidad de Cartagena Facultad de Medicina Universidad de Cartagena Cartagena de Indias Colombia; 4 Facultad de Ciencias de la Salud, Universidad Simón Bolívar, Barranquilla, Colombia Universidad Simón Bolívar Facultad de Ciencias de la Salud Universidad Simón Bolívar Barranquilla Colombia; 5 Facultad de Ciencias Biológicas, Universidad Autónoma de Nuevo León, San Nicolás de los Garza, México Universidad Autónoma de Nuevo León Facultad de Ciencias Biológicas Universidad Autónoma de Nuevo León San Nicolás de los Garza Mexico

**Keywords:** Aedes aegypti, resistencia a los insecticidas, insecticidas organofosforados, temefos, malatión, Colombia, Aedes aegypti, insecticide resistance, insecticides, organophosphate, temefos, malathion, Colombia

## Abstract

**Introducción.:**

El dengue es un problema de salud pública para el departamento de La Guajira. El control se ha enfocado en el vector con el uso de insecticidas, entre ellos los organofosforados.

**Objetivo.:**

Evaluar el estado de la sensibilidad a insecticidas organofosforados de quince poblaciones de *Aedes aegypti* (L.) en el departamento de La Guajira, Colombia.

**Materiales y métodos.:**

Se realizaron bioensayos para temefos, malatión y pirimifos- metil en larvas de tercer estadio y mosquitos adultos de *Ae. aegypti* en los municipios de Albania, Barrancas, Dibulla, Distracción, El Molino, Fonseca, Hatonuevo, La Jagua del Pilar, Maicao, Manaure, Riohacha, San Juan del Cesar, Uribia, Urumita y Villanueva, siguiendo la metodología de la Organización Mundial de la Salud (OMS) y la técnica de botellas usando la guía de los de los *Centers for Disease Control and Prevention*, respectivamente. Se determinó la sensibilidad por medio de la relación de resistencia a CL_50_ y CL_95_ (RRCL_50_, RRCL_95_) para temefos y a dosis y tiempo diagnóstico para temefos, malatión y pirimifos-metil en las poblaciones de campo evaluadas, usando como control la cepa sensible Rockefeller.

**Resultados.:**

Las 15 poblaciones del departamento de La Guajira son sensibles a: temefos (relación de la resistencia a CL_50_<5,0; relación de resistencia a CL_95_<5,0; 98 a 100 % de mortalidad); pirimifos-metil (99 a 100 % de mortalidad) y malatión (100 % de mortalidad).

**Conclusión.:**

Con base en los resultados obtenidos, es factible el uso de temefos, malatión y pirimifos-metil para el control de *Ae. aegypti* en las poblaciones evaluadas.

## Abstract (en)

**Introduction.:**

Dengue is a public health problem in La Guajira region. Control has focused on the vector using insecticides, including organophosphates.

**Objective.:**

To evaluate the state of susceptibility to organophosphates insecticides in fifteen *Aedes aegypti* (L.) populations in La Guajira, Colombia.

**Materials and methods.:**

We collected samples of third-instar larvae and adult mosquitoes of *Ae. aegypti* in the municipalities of Albania, Barrancas, Dibulla, Distracción, El Molino, Fonseca, Hatonuevo, La Jagua del Pilar, Maicao, Manaure, Riohacha, San Juan del Cesar, Uribia, Urumita, Villanueva. Bioassays for temefos, malathion, and pirimiphos-methyl were carried out following the methodology of the World Health Organization, and the bottle technique using the guidance of the Centers for Disease Control and Prevention. Susceptibility to temefos was determined through the resistance ratio between lethal concentration 50 and lethal concentration 95; for the compounds temefos, malathion and pirimiphos-methyl, susceptibility was calculated using diagnostic dose and diagnostic time in the populations evaluated. Rockefeller susceptible strain was used as a control.

**Results:**

All evaluated populations of *Ae. aegypti* from La Guajira were found to be susceptible to temefos (ratio resistance to CL_50_<5.0; ratio resistance to CL_95_<5.0; 98 - 100 % mortality); pirimiphosmethyl (99 - 100 % mortality), and malathion (100 % mortality).

**Conclusion.:**

Based on the results, the use of temefos, malathion, and pirimiphosmethyl is feasible for the control of *Ae. aegypti* in the evaluated populations.

*Aedes aegypti* (L.) (Diptera: Culicidae) es una especie de interés en salud pública por ser vector de los virus del dengue, del chikungunya, del Zika y de la fiebre amarilla ^1^. Estas arbovirosis tienen un alto impacto en el continente americano por su tasa de morbimortalidad. Particularmente, la incidencia del dengue tiende a incrementarse en los países de la región al pasar de 1,5 millones de casos acumulados en la década de los 80 a 16,2 millones de casos en la década comprendida entre 2010 y 2019 ^2^.

Colombia es uno de los países en América con mayor incidencia de dengue ^3^, con un comportamiento creciente en el número de casos: pasó de 272.360 casos acumulados en la década de los 90 a 999.102 casos entre el 2010 y 2021 ^4^^-^^8^.

En el departamento de La Guajira, el dengue mantiene un comportamiento endemo-epidémico con una incidencia acumulada de 6.346 casos, aproximadamente, en el período 2015 a 2021. Los municipios con mayor incidencia de dengue por cada 100.000 habitantes en el departamento son: Riohacha (477,0), Dibulla (318,0), Uribía (281,8) y Manaure (275,1). Sumado a lo anterior, entre los años 2014 a 2015 se introdujeron los virus del chikungunya y del Zika. Se notificaron 221 casos de chikungunya y 741 de Zika en el período 2015 a 2021 ^9^.

Para el control de estas enfermedades, durante brotes o epidemias, se han usado por más de tres décadas los compuestos organofosforados como el malatión y el temefos; desde la década de los 90, los piretroides como la lambdacialotrina y la deltametrina; y en los últimos cinco años, la alfacipermetrina. Con el tiempo, la presión de selección ejercida por los insecticidas ha generado poblaciones resistentes a los organoclorados como el dicloro-difenil-tricloroetano y a los piretroides en los municipios de San Juan del Cesar y Riohacha. Se ha reportado que esta resistencia, tipo *knockdown,* está asociada a mutaciones en la secuencia de los canales de sodio dependientes de voltaje ^10^^,^^11^.

Para el caso de los organofosforados solo existen antecedentes de sensibilidad a temefos, malatión y pirimifos-metil en los municipios de San Juan del Cesar, Maicao y Riohacha, y para fenitrotión en San Juan del Cesar ^12^.

Es importante aclarar que estos reportes datan de hace 10 años, por lo que se desconoce el estado actual de la sensibilidad de estas tres poblaciones y no existe línea base en las poblaciones de los demás municipios del departamento de La Guajira. Lo anterior es importante para la toma de decisiones en la prevención y control de las arbovirosis mencionadas, por parte de las secretarías de salud departamentales y municipales.

Teniendo en cuenta lo anterior, el objetivo del presente estudio fue evaluar el estado actual de la sensibilidad a insecticidas organofosforados de las poblaciones de *Ae. aegypti* del departamento de La Guajira, Colombia.

## Materiales y métodos

### Área de estudio

El estudio se realizó en la región caribe colombiana, específicamente en el departamento de La Guajira entre los años 2020 y 2022. El departamento de La Guajira está localizado entre los 10° 23’ y 12° 28’ de latitud norte y los 71° 06’ y 73° 39’ de longitud oeste. Limita por el norte con el mar Caribe, por el este con el mar Caribe y la República de Venezuela, por el sur con el departamento del Cesar y por el oeste con el departamento del Magdalena y el mar Caribe.

**Figura 1:**
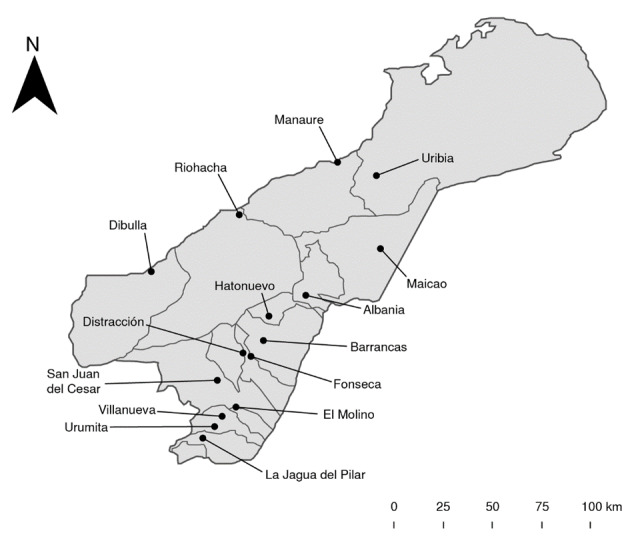
Municipios en el departamento de La Guajira (Colombia), donde se realizó la recolección de *Aedes aegypti*.

El estudio incluyó los 15 municipios que conforman el departamento de La Guajira: Albania, Barrancas, Dibulla, Distracción, El Molino, Fonseca, Hatonuevo, La Jagua del Pilar, Maicao, Manaure, Riohacha, San Juan del Cesar, Uribia, Urumita y Villanueva (figura 1).

Los criterios para la selección de los barrios donde se recolectaron las formas inmaduras fueron: la alta incidencia de dengue, chikungunya y Zika, de acuerdo con la información epidemiológica disponible de las variables de tiempo, lugar y persona, notificadas en el Sistema de Vigilancia Epidemiológica Nacional del Instituto Nacional de Salud de Colombia; la frecuencia de aplicación de insecticidas para control vectorial durante brotes -en adultos y en formas inmaduras- y los altos índices de *Aedes* adultos por vivienda (mayor de 15 %), depósitos (mayor de 10 %) y de insectos en desarrollo (índice de Breteau mayor de 25%), en los últimos tres años previos al inicio de la investigación ^12^.

## Recolección de cepas parentales y obtención de la generación filial F_1_ de *Aedes aegypti*

La recolección del material entomológico se realizó en viviendas de cada uno de los barrios seleccionados bajo un esquema de muestreo aleatorio por conglomerado. El muestreo se realizó una vez por municipio e incluyó una muestra representativa de las viviendas. Estas se incluyeron si al momento de la inspección entomológica, al menos, un residente adulto se encontraba presente y permitía el ingreso para la inspección.

En cada una de ellas se recolectaron formas inmaduras de *Ae. aegypti* en criaderos como albercas, canecas plásticas o metálicas, llantas y floreros, entre otros. El material entomológico recolectado se transportó en frascos plásticos de 2 litros al insectario de la Universidad Libre, seccional Barranquilla. Allí se obtuvo la generación F_1_ y con ella se realizaron los bioensayos bajo condiciones controladas de temperatura (28 °C ± 2 °C), humedad relativa (60 % ± 10 %) y fotoperíodo (12 horas luz:12 horas oscuridad).

### Bioensayos

Se evaluaron tres insecticidas organofosforados: temefos, malatión y pirimifos-metil. A partir de la solución original, grado técnico, de cada insecticida (ChemService, West Chester, PA), se prepararon soluciones de trabajo de cada una de las concentraciones por evaluar utilizando como diluyente etanol absoluto.

Los bioensayos para temefos se realizaron siguiendo la metodología recomendada por la OMS (1981) ^13^. Para la evaluación de la sensibilidad se emplearon larvas de tercer estadio de cada una de las 15 poblaciones, así como de la cepa de referencia Rockefeller. Se hicieron cuatro repeticiones más un control. En cada repetición se expusieron entre 15 y 25 larvas. En los tratamientos se utilizó la concentración diagnóstica de 0,012 ppm para temefos con un tiempo de exposición de 24 horas y en los controles se agregó etanol absoluto (Merck™).

Además de la dosis diagnóstica para temefos, se evaluó la sensibilidad mediante la relación de resistencia para establecer la línea base y comparar en el tiempo el estado de la sensibilidad a este larvicida en el departamento de La Guajira. Para esto se evaluaron entre cinco y ocho concentraciones de temefos en el rango de 0,01 a 0,06 pg/ml para las poblaciones recolectadas y para la cepa Rockefeller entre 0,004 y 0,015 pg/ml. Se obtuvieron mortalidades entre el 2 y el 98 %. Se hicieron tres repeticiones por cada concentración evaluada con sus respectivos controles. En cada una de ellas se expusieron entre 15 y 25 larvas de tercer estadio y se hizo la lectura de mortalidad a las 24 horas después de la exposición.

Los bioensayos para mosquitos adultos con malatión y pirimifos-metil se realizaron siguiendo la técnica de botellas según la guía de los *Centers for Disease Control and Prevention* (CDC) ^14^.

Se emplearon hembras de la F_1_ de tres días de nacidas, sin alimentar, de cada una de las poblaciones evaluadas y de la cepa Rockefeller como grupo control. Cada bioensayo estuvo conformado por tres botellas de vidrio Schott™ de 250 ml que fueron impregnadas previamente con 1 ml de la dosis diagnóstica de los insecticidas evaluados: 50 pg de malatión por botella, 75 pg de pirimifos-metil por botella, y una botella control impregnada previamente con 1 ml de etanol absoluto. En cada una de las botellas se expusieron entre 15 y 25 hembras adultas. La mortalidad para malatión y pirimifos-metil se evaluó en el tiempo diagnóstico de 30 minutos para malatión, según lo establecido por el CDC (14) y de 45 minutos para pirimifos-metil, según lo reportado por el Instituto Nacional de Salud de Colombia ^15^^,^^16^.

En todos los bioensayos se realizó corrección por fórmula de Abbott (1925), cuando se encontraron mortalidades entre el 5 y el 20 % para el control y se invalidó el bioensayo cuando la mortalidad superó el 20% ^17^. 

### Análisis de resultados

Los resultados de los bioensayos con dosis diagnósticas para temefos, malatión y pirimifos-metil se interpretaron de acuerdo con los criterios definidos por la OMS (2016) ^18^: con mortalidades mayores o iguales al 98 %, la población es sensible; entre 90 y 97 %, hay posible resistencia, e inferiores al 90 %, hay resistencia confirmada.

A partir de las concentraciones evaluadas y los porcentajes de mortalidad obtenidos para temefos, se realizó un análisis de regresión tipo *probit*^17^ empleando el programa estadístico SPSS™, versión 25. Se determinó la concentración letal 50 (CL_50_) y la concentración letal 95 (CL_95_) para temefos en cada población evaluada.

Finalmente, se calculó la relación de resistencia a CL_50_ y a CL_95_, a las 24 horas después de la exposición, al dividir el resultado de la CL_50_y CL_95_ de cada población entre la CL_50_ y CL_95_ de la cepa Rockefeller. En cada uno de los casos, el resultado de la relación de resistencia se interpretó de acuerdo con el criterio propuesto por Mazarri y Georghiou ^19^: sensible (<5X), resistencia moderada (5 a 10X), resistencia alta (>10X).

## Resultados

**Figura 2:**
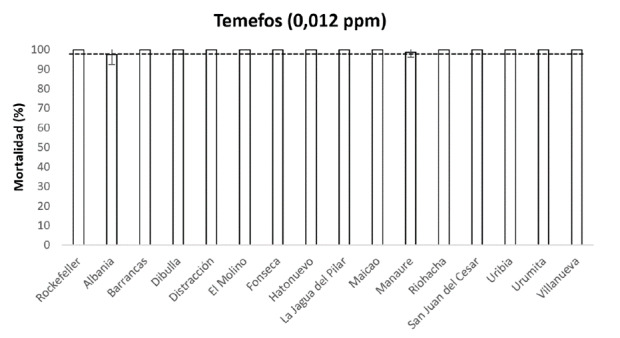
Mortalidad evaluada con una dosis de 0,012 ppm y 24 horas de exposición a temefos en poblaciones de *Aedes aegypti* del departamento de La Guajira, Colombia

Todas las poblaciones evaluadas fueron sensibles a temefos tanto por dosis diagnóstica (98 a 100 % de mortalidad) (figura 2), como por relación de resistencia (RRCL_50_<5,0; RRCL_95_<5,0) (cuadro 1). Resultados similares se encontraron con la dosis y el tiempo diagnóstico evaluado para pirimifos-metil (99 a 100 % de mortalidad) y malatión (100% de mortalidad) (cuadro 2).

**Cuadro 1:** Concentración letal 50 (CL_50_), concentración letal 95 (CL_95_) y relación de resistencia (RRCL_50_), (RRCL_95_) para temefos en poblaciones de *Aedes aegypti* del departamento de La Guajira, Colombia

Población	n	CL_50_ (ppm) (IC_95_%)	CL_95_ (ppm) (IC_95_%)	b(±ES)	x^2^ (gl)	^*p*^	RR	
							CL_50_	CL_95_
Rockefeller	473	0,001 (0,001-0,001)	0,002 (0,002-0,004)	4,505 (0,441)	17,074 (6)	0,000	1,0	1,0
Albania	445	0,003 (0,003-0,003)	0,005 (0,005-0,005)	8,765 (0,650)	8,477 (6)	0,000	3,0	2,5
Barrancas	466	0,002 (0,002-0,002)	0,003 (0,003-0,003)	9,750 (0,785)	4,451 (6)	0,000	2,0	1,5
Dibulla	432	0,003 (0,003-0,004)	0,005 (0,005-0,008)	8,206 (0,754)	20,105 (5)	0,000	3,0	2,5
Distracción	353	0,003 (0,002-0,004)	0,006 (0,004-0,060)	7,269 (0,759)	32,798 (4)	0,000	3,0	3,0
El Molino	393	0,003 (0,002-0,003)	0,005 (0,004-0,006)	7,237 (0,628)	13,312 (5)	0,000	3,0	2,5
Fonseca	362	0,003 (0,003-0,004)	0,005 (0,004-0,009)	9,830 (1,061)	20,403 (4)	0,000	3,0	2,5
Hatonuevo	340	0,003 (0,002-0,004)	0,005 (0,004-0,051)	6,222 (0,672)	27,198 (4)	0,000	3,0	2,5
La Jagua del Pilar	323	0,002 (0,002-0,002)	0,005 (0,004-0,011)	4,111 (0,413)	10,618 (4)	0,000	2,0	2,5
Maicao	340	0,003 (0,002-0,003)	0,004 (0,003-0,006)	8,655 (0,859)	13,663 (4)	0,000	3,0	2,0
Manaure	285	0,003 (0,002-0,003)	0,005 (0,004-0,017)	6,088 (0,783)	8,253 (3)	0,000	3,0	2,5
Riohacha	411	0,004 (0,003-0,005)	0,009 (0,007-0,023)	4,414 (0,540)	13,420 (5)	0,000	4,0	4,5
San Juan del Cesar	362	0,003 (0,003-0,003)	0,005 (0,004-0,005)	10,439 (0,895)	6,251 (4)	0,000	3,0	2,5
Uribia	276	0,004 (0,003-0,004)	0,006 (0,005-0,007)	8,344 (0,994)	2,588 (3)	0,000	4,0	3,0
Urumita	358	0,004 (0,004-0,004)	0,007 (0,006-0,008)	6,930 (0,681)	1,575 (4)	0,000	4,0	3,5
Villanueva	291	0,003 (0,003-0,003)	0,006 (0,005-0,009)	4,647 (0,654)	1,223 (3)	0,000	3,0	3,0

n: tamaño de la muestra (larvas evaluadas); CL_50_: concentración letal 50; CL_95_: concentración letal 95; IC_95_ %: intervalo de confianza de 95 %; RRCL_50_: relación de resistencia a las 24 horas después de la exposición entre la CL_50_ de la cepa de campo y la CL_50_ de la cepa sensible; RRCL_95_: relación de resistencia a las 24 horas después de la exposición entre la CL_95_ de la cepa de campo y la CL_95_ de la cepa sensible; b: pendiente de la regresión lineal en el modelo *probit*, en escala logarítmica; ± ES: error estándar; **x** ^2^: ji al cuadrado; gl: grados de libertad

**Cuadro 2:** Mortalidad evaluada según la dosis y el tiempo diagnóstico para malatión y pirimifos-metil en poblaciones de *Aedes aegypti* del departamento de La Guajira, Colombia

Insecticida	Población	n	Mortalidad (%)
Malatión	Rockefeller	64	100
(DD:50 μg/botella; TD: 30 minutos)	Albania	68	100
	Barrancas	63	100
	Dibulla	61	100
	Distracción	74	100
	El Molino	76	100
	Fonseca	64	100
	Hatonuevo	75	100
	La Jagua del Pilar	70	100
	Maicao	82	100
	Manaure	64	100
	Riohacha	60	100
	San Juan del Cesar	61	100
	Uribia	62	100
	Urumita	68	100
	Villanueva	68	100
Pirimifos-metil	Rockefeller	68	100
(DD: 75 μg/botella; TD: 45 minutos)	Albania	62	100
	Barrancas	67	100
	Dibulla	63	100
	Distracción	75	100
	El Molino	75	100
	Fonseca	74	100
	Hatonuevo	91	100
	La Jagua del Pilar	77	100
	Maicao	84	99
	Manaure	65	100
	Riohacha	76	100
	San Juan del Cesar	66	100
	Uribia	64	100
	Urumita	72	100
	Villanueva	68	100

n: tamaño de la muestra (hembras evaluadas); DD: dosis diagnóstica; TD: tiempo diagnóstico

## Discusión

Los resultados del presente trabajo confirman la sensibilidad a insecticidas organofosforados reportada previamente en los municipios de Maicao, San Juan del Cesar y el distrito de Riohacha, y amplían la información sobre el estado de sensibilidad a organofosforados en los demás municipios del departamento de La Guajira donde no existían estudios previos. En Maicao y Riohacha se había reportado sensibilidad a temefos y malatión ^20^ y en San Juan del Cesar se había descrito a temefos, malatión, fenitrotión y pirimifos-metil ^12^.

Los hallazgos obtenidos en el presente estudio coinciden con los anteriores, lo cual indica que las poblaciones aún se mantienen sensibles a este tipo de organofosforados y que esta sensibilidad es generalizada en todo el departamento de La Guajira.

En el caso puntual del temefos, los resultados obtenidos probablemente se deben a que en el departamento de La Guajira se ha alternado el uso de este insecticida con reguladores de crecimiento, lo cual ha impactado de forma positiva al mantener la sensibilidad al temefos en estas poblaciones.

Teniendo en cuenta lo anterior, es importante mantener un uso razonable de este tipo de compuestos organofosforados y fortalecer otras estrategias departamentales para la prevención y control de las arbovirosis que pueden ser transmitidas por *Ae. aegypti*; asimismo, vigilar en el tiempo la sensibilidad a estos insecticidas de las poblaciones evaluadas con el fin de detectar con prontitud potenciales cambios.

En la región caribe colombiana se ha reportado que la mayoría de las poblaciones de *Ae. aegypti* son sensibles a insecticidas organofosforados ^12^. Puntualmente, para el temefos se ha reportado sensibilidad en algunas poblaciones de *Ae. aegypti* de los departamentos de Atlántico, Cesar, Sucre, Magdalena, Córdoba y Bolívar ^12^. Sin embargo, se han registrado poblaciones resistentes a temefos y fenitrotión en los municipios de Puerto Colombia y Soledad en el departamento de Atlántico ^21^^,^^22^.

Para el pirimifos-metil solo se tiene antecedente de resistencia en el departamento de Sucre ^12^. En cuanto a las poblaciones de *Ae. aegypti* de las demás regiones del país, se ha reportado sensibilidad a temefos en algunas poblaciones de Santander, Caldas y Casanare ^16^^,^^23^^,^^24^ y resistencia a este larvicida y al fenitrotión en los departamentos de Cauca, Nariño, Huila y Valle del Cauca ^15^^,^^25^; resistencia al temefos en Cundinamarca, Guaviare, Meta, Santander y Norte de Santander ^16^^,^^26^; y a pirimifos-metil en Caldas ^23^. Es importante resaltar que en las poblaciones de *Ae. aegypti* de estos y otros departamentos de Colombia se ha reportado sensibilidad al malatión ^23^ de igual forma a como se encontró en las poblaciones analizadas en el departamento de La Guajira.

En conclusión, encontramos sensibilidad a los insecticidas organofosforados evaluados en todas las poblaciones de *Ae. aegypti* muestreadas en el departamento de La Guajira, por lo que es factible el uso de temefos, malatión y pirimifos-metil para el control de esta especie de mosquito en esta región de Colombia.
